# Regional emphysema score is associated with tumor location and poor prognosis in completely resected NSCLC patients

**DOI:** 10.1186/s12890-020-01268-7

**Published:** 2020-09-11

**Authors:** Jung Won Heo, Hye Seon Kang, Chan Kwon Park, Sung Kyoung Kim, Ju Sang Kim, Jin Woo Kim, Seung Joon Kim, Sang Haak Lee, Chang Dong Yeo

**Affiliations:** 1 Division of Pulmonary, Critical Care and Allergy, Department of Internal Medicine, Eunpyeong St. Mary’s Hospital, College of Medicine, The Catholic University of Korea, 1021, Tongil-ro, Eunpyeong-gu, Seoul, 03312 Republic of Korea; 2 Division of Pulmonary, Critical Care and Allergy, Department of Internal Medicine, Bucheon St. Mary’s Hospital, College of Medicine, The Catholic University of Korea, Seoul, Republic of Korea; 3 Division of Pulmonary, Critical Care and Allergy, Department of Internal Medicine, Yeouido St. Mary’s Hospital, College of Medicine, The Catholic University of Korea, Seoul, Republic of Korea; 4 Division of Pulmonary, Critical Care and Allergy, Department of Internal Medicine, St. Vincent’s Hospital, College of Medicine, The Catholic University of Korea, Seoul, Republic of Korea; 5 Division of Pulmonary, Critical Care and Allergy, Department of Internal Medicine, Incheon St. Mary’s Hospital, College of Medicine, The Catholic University of Korea, Seoul, Republic of Korea; 6 Division of Pulmonary, Critical Care and Allergy, Department of Internal Medicine, Uijeongbu St. Mary’s Hospital, College of Medicine, The Catholic University of Korea, Seoul, Republic of Korea; 7 Division of Pulmonary, Critical Care and Allergy, Department of Internal Medicine, Seoul St. Mary’s Hospital, College of Medicine, The Catholic University of Korea, Seoul, Republic of Korea

**Keywords:** Lung cancer, Location, Prognosis, Regional emphysema, Visual score

## Abstract

**Background:**

Lung cancer is a frequent comorbidity of chronic obstructive pulmonary disease (COPD). However, the local risk of developing lung cancer related to regional emphysema distribution and clinical outcome has not been investigated. Our aim was to evaluate the impact of regional emphysema score (RES) on tumor location and prognosis in non-small cell lung cancer (NSCLC) patients.

**Methods:**

We enrolled 457 patients who underwent curative surgery for NSCLC at seven hospitals at The Catholic University of Korea from 2014 to 2018. Emphysema was visually assessed for each lobe, with the lingula as a separate lobe. Semi-quantitative emphysema scoring was classified as follows: 0 = none, 0.5 = 1 to 10%, 1 = 11 to 25%, 2 = 26 to 50%, 3 = 51 to 75%, and 4 = 76 to 100%. An RES was given to each of the six lung zone: the upper, middle, and lower lobes in the right and left lungs.

**Results:**

There were 145 patients in the high RES (≥ 3) group and 312 in the low RES (< 3) group. The mean RES in each lobe with cancer was significantly higher than that in other lobes without cancer (0.51 vs. 0.37, *P* <  0.001). This group showed significantly shorter disease-free survival (*P* <  0.001), in addition, presence of COPD, low diffusing capacity of the lung for carbon monoxide (< 80), smoking status, and poor differentiation were more frequent in this group. Also, cancer in a lobe with a higher RES (odds ratio (OR) = 1.56; 95% confidence interval (CI:1.01–2.42; *P* = 0.04), pathologic stage ≥ III (OR = 2.23; 95% CI: 1.28–3.89; *P* <  0.001), and poor differentiation (OR = 1.99; 95% CI: 1.22–3.21; *P* <  0.001) were independent factors for tumor recurrence.

**Conclusions:**

The regional severity of emphysema by visual qualification was associated with the location of lung cancer, and was an independently poor prognostic factor for tumor recurrence in completely resected NSCLC patients.

## Background

Chronic obstructive pulmonary disease (COPD) and lung cancer are major causes of death worldwide [1]. COPD is a well-known risk factor for the development of lung cancer. The cause of death reported in patients with COPD varies significantly among studies, but the mortality from lung cancer is relatively high [2]. Emphysema is one of the subtypes of COPD, characterized by parenchymal destruction [3]. Among several possible explanations for a relationship between emphysema and lung cancer, the most likely is due to a shared causal pathway in lung and airway inflammation [4, 5]. Tobacco smoke, a major risk factor for emphysema and lung cancer, is known to stimulate airway inflammation [6]. Chronic inflammation has been implicated in the pathogenesis of many cancers, including lung cancer [7]. The injury and repair can be repeated, causing cell turnover and potential genetic errors, ultimately resulting in the development of lung cancer [8].

Emphysema is often observed heterogeneously, and computed tomography (CT) is the standard method for non-invasive diagnosis and quantifying emphysema [9]. According to some studies, in most patients, emphysema was predominant in the upper lung [10]. Nemec et al. [11] reported that mild and moderate centrilobular emphysemas produced multiple small, round areas of low attenuation, usually in the upper lobe. In contrast, panlobular emphysema produces uniform destruction of the secondary lobule, which results in homogenous low attenuation that may involve the entire lung. Recently, several studies have reported that the severity of emphysema on CT was associated with the presence of lung cancer [12, 13]. In the degree of COPD severity, visual emphysema, as well as the airway obstruction of lung function, were independent predictors of lung cancer [14]. Also, cancer was much more likely to develop in the area of the lung with the highest emphysema score in each individual [15].

As such, there have been studies on the association between regional emphysema severity and lung cancer, but the prognostic role of emphysema score of specific lobe with lung cancer remains unclear. Based on the heterogeneous distribution of emphysema, this study investigated the impact of the regional emphysema score (RES) and tumor location and prognosis in completely resected non-small cell lung cancer (NSCLC) patients.

## Methods

### Data source

We enrolled completely resected NSCLC patients at seven medical centers in the Catholic University of Korea from January 2014 to December 2018. The Catholic Medical Center (CMC) lung cancer registry consists of seven multi-centers (Seoul St. Mary’s Hospital, Yeouido St. Mary’s Hospital, Eunpyeong St. Mary’s Hospital, Uijeongbu St. Mary’s Hospital, Bucheon St. Mary’s Hospital, Incheon St. Mary’s Hospital, and St. Vincent’s Hospital) in capital region of South Korea. Data were collected on basic demographics, which included age, gender, Eastern Cooperative Oncology Group (ECOG) performance status, smoking history, pathologic staging, differentiation, epidermal growth factor receptor (EGFR) mutations and anaplastic lymphoma kinase (ALK) rearrangement, percent forced expiratory volume in one second (FEV1%), obstructive lung disease, diffusing capacity of the lung for carbon monoxide (DLCO), extent of lung resection, as well as pleural, lymphatic, and vascular invasion. In our data, smoking history included smoking status, pack-years, and the duration of smoking cessation. We defined ever-smokers as those who smoked at least 100 cigarettes over the course of their lifetime. Patients who had smoked fewer than 100 cigarettes in their lifetime were defined as never-smokers [16]. The lung function tests were performed by the American Thoracic Society/European Respiratory Society standardization guidelines. The inclusion criteria were a diagnosis of COPD by a pulmonologist; age ≥ 40 years; symptoms, including cough, sputum, dyspnea; and a post-bronchodilator FEV1/forced vital capacity (FVC) of 70% less than the normal predicted value. TNM stage was classified according to the 8th American Joint Committee on Cancer tumor, node, and metastasis classification. The recorded pathological variables included tumor size, the tumor differential grade, visceral pleural invasion, angiolymphatic invasion, tumor necrosis, tumor histology, and lymph node dissection numbers. Patients above stage II received adjuvant chemotherapy. We divided extent of lung resection into sublobar resection, lobectomy, and pneumonectomy in all patients. A cancer recurrence is defined as cancer that relapses either radiographically or histologically after treatment and following a period of time in which there is no evidence of cancer [17]. Disease-free survival was defined as the time from surgery to recurrence or death. By qualified data managers, clinical information, including stage, pathology, treatment modality and survival, were systematically recorded to improve the accuracy of the data. This study was approved by the Clinical Research Ethics Committee of the Catholic Medical Center (XC140IMI0070).

### Visual emphysema score

Emphysema evaluation was based on a standard-dose CT scan before surgery. Emphysema was characterized by CT areas of low attenuation surrounded by normal lung attenuation. The presence of emphysema was visually assessed for each lobe, with the lingula as a separate lobe. The severity of emphysema was visually evaluated with the modified Goddard scoring system by two or more experienced pulmonologists and blinded to the patients’ clinical data [9, 18]. Semi-quantitative emphysema scoring was classified as follows: 0 = none, 0.5 = 1 to 10%, 1 = 11 to 25%, 2 = 26 to 50%, 3 = 51 to 75%, and 4 = 76 to 100%. An RES was given to each of six lung zones, the upper, middle (or lingula), and lower lobes in the right and left lungs [9]. Therefore, the total RESs ranged from 0 to a maximum of 24. High RES was defined by 3 or more of the total 24 points. The patients were then divided into two groups according to emphysema score of each lobe with/without cancer, one for cancer in lobe with a higher RES than non-cancer lobes and the other for cancer in a lobe with a lower RES than non-cancer lobes.

### Defining cutoff values

Receiver operating characteristic (ROC) curve was generated for RES to determine the cut-off value for predicting disease free survival yielded optimal sensitivity and specificity. The patients were then allocated to high/low groups based on the cut-off values.

### Statistical analysis

The clinical data were compared using Pearson’s chi-squared tests and unpaired *t*-tests for continuous variables. Unpaired t-tests were used to analyze the association between the emphysema scores and tumor location. Survival curves according to the prognostic factors were drawn using the Kaplan–Meier method and survival differences were analyzed by the log-rank test. The Cox proportional hazards modeling technique was applied to identify the independent prognostic factors. Hazard ratios (HRs) and corresponding 95% confidence intervals (CIs) were calculated for predictors that were significant in multivariate analysis. A two-sided *P* value < 0.05 was considered to be statistically significant. All statistical analyses were performed using SAS version 9.4 (SAS Institute Inc., Cary, NC, USA).

## Results

### Clinical characteristics according to RES

The baseline characteristics of the two groups are summarized in Table 1. A total of 457 patients were divided into high RES and low RES groups based on three points. The reason we set the cut-off level of RES to 3 in this study was based on the ROC curve. The area under the curve of the ROC curve for RES was 0.797, and the *P* value was statistically significant as < 0.001. We set the cut-off level of 3, which is between 2.75 and 3.25, which was the point of high sensitivity and specificity for RES. There were 145 patients in the high RES (≥ 3) group and 312 in the low RES (< 3) group. The mean age of the patients in the high RES group was higher (69.86 ± 8.14 vs. 64.96 ± 9.07, *P* <  0.001). The proportion of male gender (86.9% vs. 53.5%, *P* <  0.001), ECOG ≥2 (4.8% vs. 1.0%, *P* = 0.009) and smokers (former or current smokers) (87.6% vs, 53.2%, *P* <  0.001) was higher in the high RES group. In histopathology, advanced pathologic stage (I: 54.5% vs. 66.3%, II and III: 55.5% vs. 33.7%, *P* = 0.001) and poor differentiation (21.4% vs. 10.6%, *P* = 0.002) were more frequent in the high RES group. In pulmonary function, the coexistence of COPD was significantly more frequent in patients with a high RES (61.4% vs. 22.8%, *P* <  0.001). The mean FEV1/FVC was lower in the high RES group, 0.65 ± 0.13 in the high RES group and 0.74 ± 0.09 in the low RES group (*P* <  0.001). Also, the mean FEV1 was lower in the high RES group (86.7 ± 21.4 vs. 96.4 ± 20.4, *P* <  0.001). The proportion of low DLCO (< 80) was higher in the high RES group (50.0% vs. 30.2%, *P* <  0.001). The mean DLCO was lower in the high RES group (79.7 ± 19.6 in the high RES group and 90.2 ± 19.0 in low RES group, *P* <  0.001).

**Table 1:** Characteristics of patients according to the emphysema score on CT (defined as a CT-based emphysema score of ≥3 of total 24)

Characteristic	Low (< 3) emphysema score (*N* = 312)	High (≥3) emphysema score (*N* = 145)	*P* value
Age	64.96 ± 9.07	69.86 ± 8.14	< 0.001
Male	167 (53.5)	126 (86.9)	< 0.001
ECOG PS			0.009
0–1	309 (99.0)	138 (95.2)	
≥ 2	3 (1.0)	7 (4.8)	
Smoking status			< 0.001
Never	146 (46.8)	18 (12.4)	
Ever	166 (53.2)	127 (87.6)	
Pathologic staging			0.001
I	203 (66.3)	78 (54.5)	
II + III	103 (33.7)	65 (55.5)	
Differentiation			0.002
Well + moderate	279 (89.4)	114 (78.6)	
Poorly	33 (10.6)	31 (21.4)	
Driver oncogene			
EGFR mutation	69(30.3)	29 (29.0)	0.818
ALK translocation	8 (72.7)	3 (27.3)	0.760
COPD	71 (22.8)	89 (61.4)	< 0.001
FEV1/FVC	0.74 ± 0.09	0.65 ± 0.12	< 0.001
FEV1, % pred	96.36 ± 20.38	86.67 ± 21.41	< 0.001
Low DLCO (< 80)	91 (30.2)	69 (50.0)	< 0.001
DLCO	90.16 ± 19.04	79.67 ± 19.58	< 0.001
Pleural invasion	77 (25.2)	45 (31.7)	0.149
Lymphatic invasion	94 (30.4)	47 (32.6)	0.635
Vascular invasion	47 (15.2)	28 (19.4)	0.259
Extent of lung resection			0.405
Sublobar resection	35 (11.2)	20 (13.8)	
Lobectomy	267 (85.6)	123 (84.8)	
Pneumonectomy	10 (3.2)	2 (1.4)	

Data are presented as mean ± standard deviation or number (%)

*Abbreviations*: *EGFR* epidermal growth factor receptor, *ALK* anaplastic lymphoma kinase, *COPD* chronic obstructive pulmonary disease, *CT* computed tomography, *DLCO* diffusing capacity of lung for carbon monoxide, *ECOG PS* Eastern Cooperative Oncology Group (ECOG) Performance Status, *FEV1* Forced expiratory volume in 1 second, *FVC* forced vital capacity, *pred* predictive value

### Emphysema scores and tumor location

**Fig. 1:**
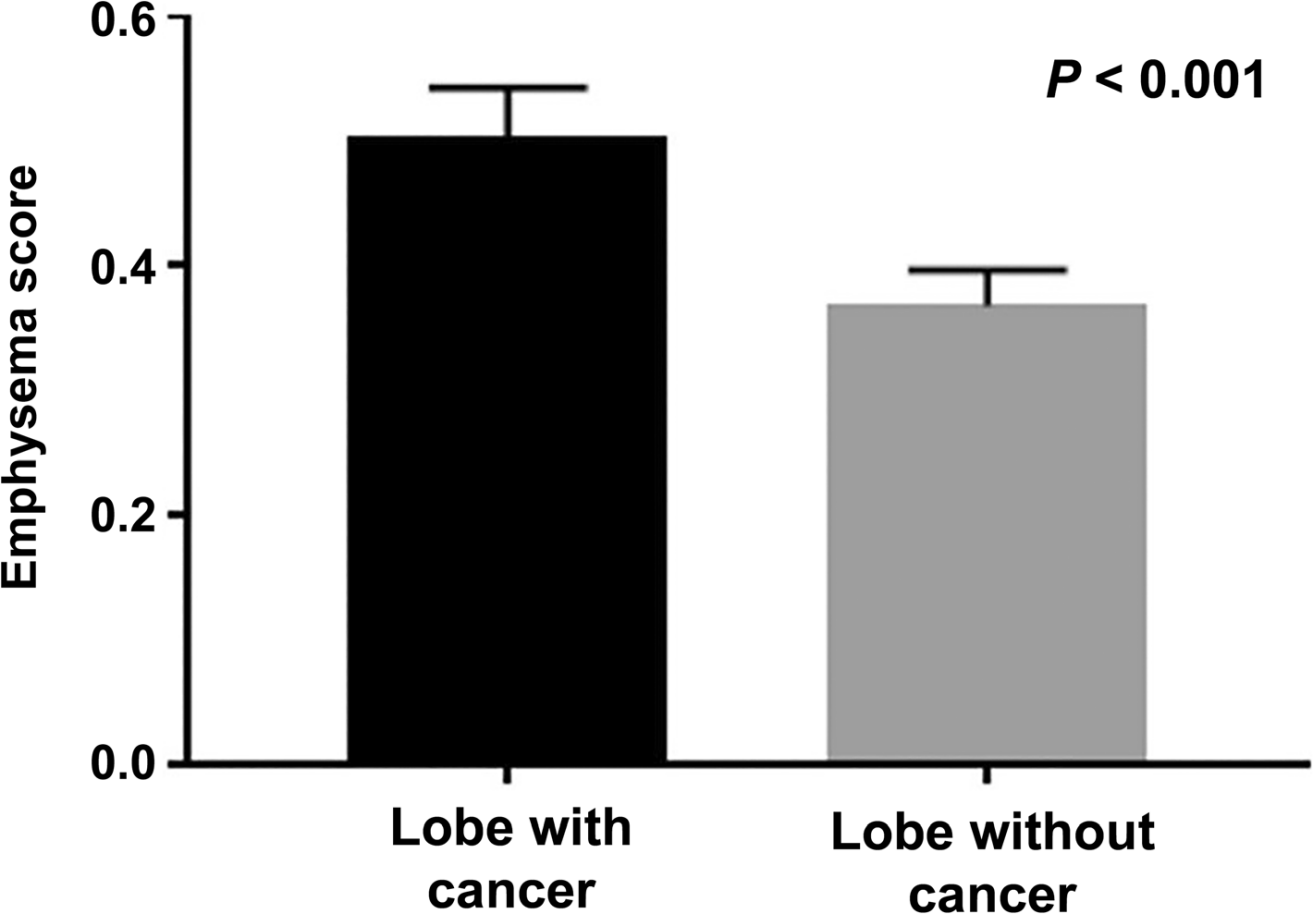
Difference of regional emphysema score between lobe with cancer and those without cancer in lung cancer patients after lobectomy (*P* < 0.001)

We analyzed the association between regional emphysema severity and tumor location. The mean RES in each lobe with cancer was statistically significantly higher than the mean of the RES in other lobes without cancer (0.51 vs. 0.37, *P* <  0.001) (Fig. 1).

Therefore, we analyzed the baseline characteristics of the group of higher RESs in cancer lobes compared to non-cancer lobes. One hundred fifty-nine (34.8%) patients had cancer in a lobe with a higher RES. COPD was frequently observed in this group (55.3% vs. 25.2%, *P* <  0.001). Cancer in a lobe with a higher RES also had a high proportion of low DLCO (< 80) (49.3% vs. 29.9%, *P* <  0.001). This group included more ever-smokers (83.0% vs. 54.0%, *P* <  0.001). In histopathology, poor differentiation (22.6% vs. 9.4%, *P* <  0.001) and pleural (34.0% vs 22.8%, *P* = 0.028) or vascular invasion (22.2% vs. 13.6%, *P* = 0.019) were frequently noted in patients with cancer in a lobe with a higher RES (Table 2).

**Table 2:** Baseline characteristics according to high or low RES in lobe with cancer

Characteristic	Cancer in a lobe with lower RES (*N* = 298)	Cancer in a lobe with higher RES (*N* = 159)	*P* value
COPD	75 (25.2)	88 (55.3)	< 0.001
DLCO (< 80)	87 (29.9)	73 (49.3)	< 0.001
Smoking			< 0.001
Never	137 (46.0)	27 (17.0)	
Ever	161 (54.0)	132 (83.0)	
Pathologic stage			0.347
I	188 (64.2)	93 (59.6)	
II	56 (19.1)	39 (25.0)	
III	49 (16.7)	24 (15.4)	
Differentiation			< 0.001
Well + Moderate	270 (90.6)	123 (77.4)	
Poorly	28 (9.4)	36 (22.6)	
Pleura invasion	68 (22.8)	54 (34.0)	0.028
Lymphatic invasion	89 (30.2)	52 (32.9)	0.548
Vascular invasion	40 (13.6)	35 (22.2)	0.019

Data are presented as number (%)

*Abbreviations*: *COPD* chronic obstructive pulmonary disease, *DLCO* diffusing capacity of lung for carbon monoxide, *RES* regional emphysema score

### Factors associated with DFS

**Fig. 2:**
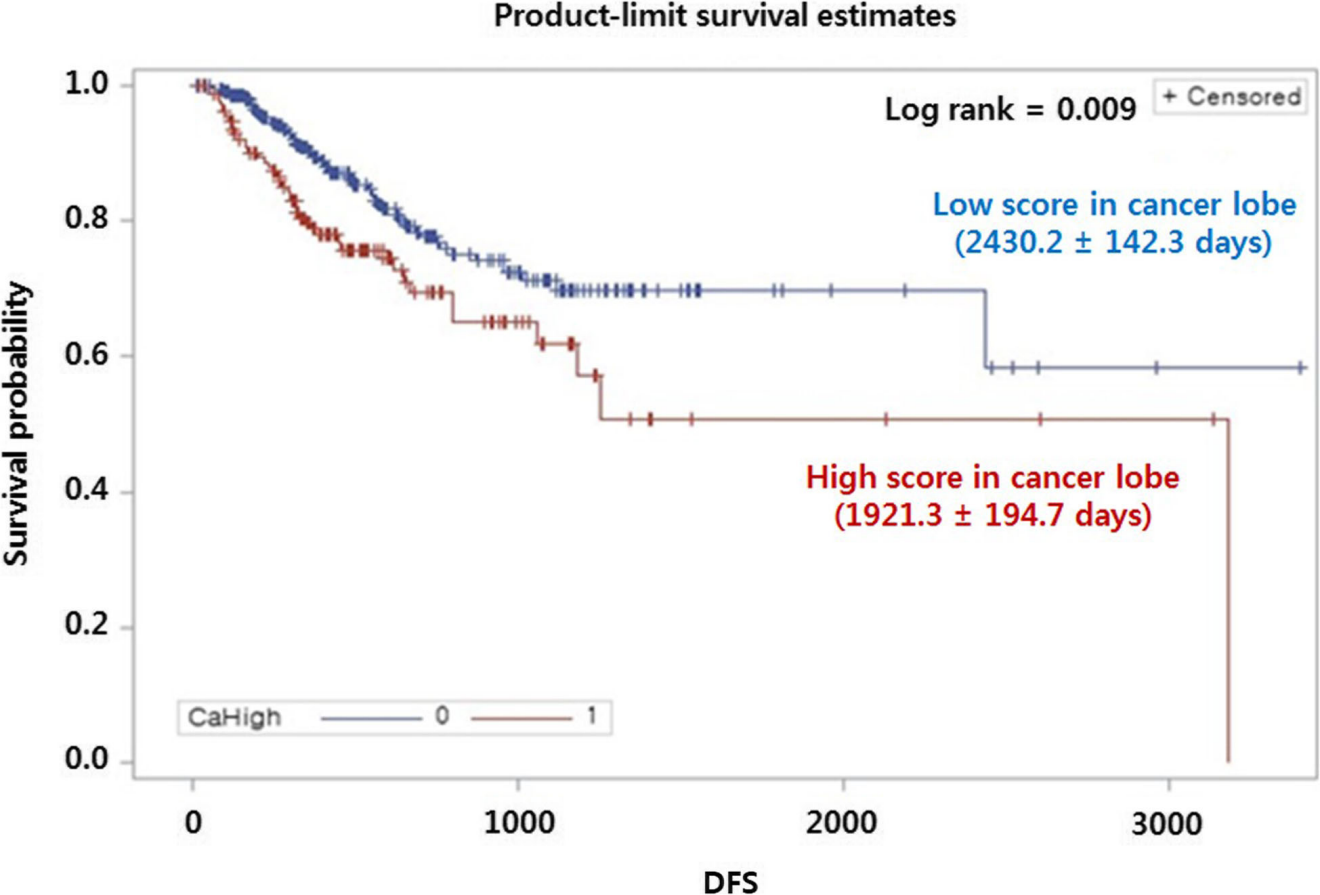
Disease free survival according to high or low regional emphysema score in lobe with cancer. Abbreviations: DFS, disease-free survival

To investigate the prognostic role of high RES in this population, we analyzed the Kaplan–Meier curve and disease-free survival (Fig. 2). Disease-free survival was significantly shorter in the cancer group with higher RESs (1921.3 ± 194.7 days vs. 2430.2 ± 142.3 days, *P* <  0.001).

In univariate Cox regression analysis for cancer recurrence, smoking (HR = 1.573; 95% CI: 1.009–2.452; *P* = 0.045), advanced pathologic staging (II: HR = 2.114; 95% CI: 1.294–3.453; *P* <  0.003, III: HR = 3.408; 95% CI: 2.123–5.468; *P* <  0.001), poor differentiation (HR = 3.221; 95% CI: 2.065–5.020; *P* <  0.001), pleural (HR = 2.313; 95% CI: 1.562–3.452; *P* <  0.001) or lymphatic (HR = 2.653; 95% CI: 1.786–3.940; *P* <  0.001) or vascular (HR = 2.600; 95% CI: 1.665–4.060; *P* <  0.001) invasion, and cancer in a lobe with a higher RES (HR = 1.698; 95% CI: 1.139–2.530; *P* < 0.001) were associated with cancer recurrence. Multivariate analysis revealed that advanced pathologic stage (HR = 2.233; 95% CI; 1.284–3.884; *P* = 0.004), poor differentiation (HR = 1.980; 95% CI: 1.223–3.206, *P* = 0.005), and cancer in a lobe with a higher RES (HR = 1.563; 95% CI: 1.011–2.416; *P* = 0.044) were independent prognostic factors for cancer recurrence (Table 3).

**Table 3:** Univariate and multivariate analysis of risk factor for lung cancer recurrence after lobectomy

Variable	Univariate		Multivariate	
	HR (95% CI)	*P* value	HR (95% CI)	*P* value
Smoking	1.573 (1.009–2.452)	0.045	1.136 (0.711–1.817)	0.594
Pathologic stage (I)				
II	2.114 (1.294–3.453)	0.003		
III	3.408 (2.123–5.468)	< 0.001	2.233 (1.284–3.884)	0.004
Poor differentiation	3.221 (2.065–5.020)	< 0.001	1.980 (1.223–3.206)	0.005
Pleural invasion	2.313 (1.562–3.452)	< 0.001	1.427 (0.925–2.202)	0.108
Lymphatic invasion	2.653 (1.786–3.94)	< 0.001	1.333 (0.801–2.216)	0.269
Vascular invasion	2.600 (1.665–4.060)	< 0.001	1.448 (0.867–2.417)	0.157
High emphysema score (≥3)	1.514 (0.999–2.295)	0.051		
Higher RES in a lobe with cancer	1.698 (1.139–2.530)	0.009	1.563 (1.011–2.416)	0.044

*Abbreviations*: *HR* hazard ratio, *CI* confidence interval, *RES* regional emphysema score

## Discussion

We showed that the location of lung cancer was significantly associated with the regional severity of emphysema. Cancer occurred in a lobe with emphysema was associated with poor pulmonary function, smoking, poorly differentiated phenotype, and extended microscopic findings. Moreover, cancer in a lobe with a higher RES was an independent poor prognostic factor of disease-free survival in completely resected NSCLC patients.

We found that higher RESs were associated with lung cancer development. This finding is further supported by other studies. Lung cancer was most common in the lung lobes with more severe emphysema [19]. Carr et al. [14] showed that visual emphysema was significantly associated with the risk of a lung cancer diagnosis. The severity of visual emphysema and the severity of obstruction as measured by FEV1% were independent predictors of lung cancer in COPD patients. Another study analyzed the frequency of RES by anatomical regions, nodules versus control regions in benign or malignant nodule cohorts [9]. In this study, malignant and benign nodule cohorts were compared, and the emphysema score was higher in malignant cohort. The correlation between regional emphysema score and nodule location was significant in malignant cohort [9, 20]. In the present study, the cancer was occurred in severe emphysema region, and the patients with higher RES had more advanced stage and more aggressive phenotype.

As a pathologic mechanism that can explain the association between emphysema and lung cancer, chronic inflammation plays an important role in the development of cancer [21]. Virchow first mentioned the relationship between cancer and inflammation, based on the presence of leukocytes in cancer tissue. He also reported that immune cells can control the stages of cancer development at all stages [22]. COPD including emphysema is a chronic inflammatory disease, in which neutrophils, monocytes, macrophages, and lymphocytes are infiltration in airway and lung tissue [23]. In addition, COPD and lung cancer are progressive diseases caused by substances that mediate this inflammatory response, which is supported by the high incidence of lung cancer in patients with severe impaired lung function and frequent exacerbations with inflammatory reactions [14]. Chronic inflammation activates inflammatory mediators such as cyclooxygenase-2, reactive nitrogen and oxygen species (RNOS), and pro-inflammatory cytokine, which breaks the balance between tumor promotion and suppression, promoting cell proliferation, angiogenesis, and genomic instability [24, 25]. Most COPD and lung cancer are mainly caused by smoking, a common epidemiologic cause. Cigarettes contain stable free radicals and RNOS [26]. RNOS damages DNA, inhibits DNA repair and apoptosis. When lung damage occurs, RNOS suppress the protective mechanism and cell proliferation occurs, resulting in carcinogenesis [27, 28]. Therefore, cancer is more likely to develop in the regions with severe emphysema through mechanisms of inflammation and free radical generation due to cigarette smoking.

Since emphysema has a heterogeneous distribution, it has been estimated that the local severity of emphysema can affect the location of lung cancer. Lobe segmentation provides more accurate information than a non-anatomic approach in identifying the impact of regional emphysema on lung cancer [29]. A few studies have shown that regional emphysema was associated with the location of lung cancer [9]. According to Bae et al. [12], more severe emphysema was found in the upper lobes and a higher frequency of lung cancer was also found in the upper lobes. In the current study, the mean RES was highest at 0.55 in the upper lobe, followed by 0.35 in the lower lobe. Lung cancer was the most common in the upper lobe (54.5%), followed by the lower lobe (37.8%), and other studies showed the similar results [29–31]. They also showed that lung cancer had more emphysema severity than lobes without cancer. Consistently with the present study, previous studies showed a significant correlation between emphysema severity and lung cancer occurrence, measured quantitatively using lobe segmentation.

In the current study, disease-free survival was significantly shorter in the cancer group with higher RESs. As factors related to the recurrence of lung cancer, several factors are known, such as tumor size, nodal metastasis, and smoking status [32–37]. Kinsey et al. [32] found that tumors occurring in regions of greater emphysema were associated with worse overall survival than tumors occurring in regions of less emphysema. We presumed that severe regional emphysema score by visual quantification associated local and systemic inflammation, which resulted in a negative effect on the development of lung cancer as well as the poor clinical outcome regarding to the cancer recurrence in resected lung cancer patients. Considering that airway inflammation has clinical importance in patients with lung cancer, presence of regional emphysema and its heterogeneous distribution are important factors to be considered in the clinical setting from lung cancer screening to surveillance after surgical resection [38, 39].

This study had some limitations. First, this study was retrospective and observational, so potential bias could not be completely eliminated. However, this was a seven multicenter study, which minimized the potential biased selection. In addition, we matched the patients who had undergone curative surgery for NSCLC to a relatively large number of control patients which provides a clearer perspective regarding prognostic factors and recurrence free-survival in completely resected NSCLC patients. Second, the emphysema can be measured by high resolution CT, using the visual score calculation, and quantitatively measuring the percentage of voxels below a certain house unit [12, 40]. Although the quantitative method of measuring emphysema has been developed, it is meaningful that we used accessible and readily available visual emphysema scoring. In addition, visual scores can capture more clinically relevant information such as spatial distribution compared with other methods [40–42]. For example, although the quantitative measures of emphysema did not show an association, the visual score was found to be associated with lung cancer development in patients with emphysema [40, 43–45]. Indeed, several COPD cohorts, including a Hokkaido cohort, score emphysema using visual quantification, suggesting that this method is relevant for clinical application.

In conclusion, the regional severity of emphysema by visual qualification was associated with the location of lung cancer, and was an independently poor prognostic factor for tumor recurrence in completely resected NSCLC patients. In addition, the cancer occurred from higher RES was associated with poor pulmonary function, smoking, poorly differentiated phenotype, and extended microscopic findings. Further studies may clarify methods of scoring emphysema and the biological mechanisms that underlie this relationship.

## Data Availability

Data analyzed in the current study are not publicly available. They may be made available from the corresponding authors upon reasonable request.

## Abbreviations

CI: Confidence interval
COPD: Chronic obstructive pulmonary disease
CT: Computed tomography
DLCO: Diffusing capacity of the lung for carbon monoxide
ECOG PS: Eastern Cooperative Oncology Group Performance Status
EGFR: Epidermal growth factor receptor
ALK: Anaplastic lymphoma kinase
FEV1: Forced expiratory volume in one second
FVC: Forced vital capacity
HRs: Hazard ratios
NSCLC: Non-small cell lung cancer
OR: Odds ratio
RES: Regional emphysema score
ROC: Receiver operating characteristic
RNOS: Reactive nitrogen and oxygen species
